# A prospective, randomized, placebo-controlled, double-blind, multicenter study of the effects of irbesartan on aortic dilatation in Marfan syndrome (AIMS trial): study protocol

**DOI:** 10.1186/1745-6215-14-408

**Published:** 2013-12-01

**Authors:** Michael J Mullen, Marcus D Flather, Xu Yu Jin, William G Newman, Guliz Erdem, David Gaze, Oswaldo Valencia, Winston Banya, Claire E Foley, Anne Child

**Affiliations:** 1 The Heart Hospital, University College London Hospitals NHS Foundation Trust, 16-18 Westmoreland Street, London W1G 8PH, UK; 2 Norwich Medical School, Norfolk and Norwich University Hospitals NHS Foundation Trust, Faculty of Medicine and Health Sciences, University of East Anglia, Norwich NR4 7TJ, UK; 3 John Radcliffe Hospital, Oxford University Hospitals NHS Trust, Headley Way, Headington, Oxford OX3 9DU, UK; 4 Genetic Medicine, St Mary’s Hospital, Central Manchester University Hospitals NHS Foundation Trust, Oxford Road, Manchester M13 9WL, UK; 5 Clinical Trials and Evaluation Unit (CTEU), Royal Brompton and Harefield NHS Foundation Trust, Royal Brompton Hospital, Sydney Street, London SW3 6NP, UK; 6 St George’s, University of London, Cranmer Terrace, London SW17 0RE, UK

## Abstract

**Background:**

Cardiovascular complications are the leading cause of mortality and morbidity in Marfan syndrome (MFS), a dominantly inherited disorder caused by mutations in the gene that encodes fibrillin-1. There are approximately 18,000 patients in the UK with MFS. Current treatment includes careful follow-up, beta blockers, and prophylactic surgical intervention; however, there is no known treatment which effectively prevents the rate of aortic dilatation in MFS. Preclinical, neonatal, and pediatric studies have indicated that angiotensin receptor blockers (ARBs) may reduce the rate of aortic dilatation. This trial will investigate the effects of irbesartan on aortic dilatation in Marfan syndrome.

**Methods/Design:**

The Aortic Irbesartan Marfan Study (AIMS) is an investigator-led, prospective, randomized, placebo-controlled, double-blind, phase III, multicenter trial. Currently, 26 centers in the UK will recruit 490 clinically confirmed MFS patients (aged ≥6 to ≤40 years) using the revised Ghent diagnostic criteria. Patients will be randomized to irbesartan or placebo. Aortic root dilatation will be measured by transthoracic echocardiography at baseline and annually thereafter. The primary outcome is the absolute change in aortic root diameter per year measured by echocardiography. The follow-up period will be a minimum of 36 months with an expected mean follow-up period of 48 months.

**Discussion:**

This is the first clinical trial to evaluate the ARB irbesartan versus placebo in reducing the rate of aortic root dilatation in MFS. Not only will this provide useful information on the safety and efficacy of ARBs in MFS, it will also provide a rationale basis for potentially lifesaving therapy for MFS patients.

**Trial registration:**

ISRCTN, 90011794

## Background

Cardiovascular complications are the leading cause of mortality and morbidity in Marfan syndrome (MFS), a dominantly inherited disorder caused by mutations in the gene that encodes fibrillin-1. There are approximately 18,000 patients in the UK with MFS. MFS is diagnosed clinically using the Ghent criteria which emphasizes the identification of a positive family history, ectopia lentis, aortic root dilatation Z-score >2, and a systemic score of clinical features [1,2]. Twenty-five percent of cases are the result of a new mutation in the fibrillin-1 gene, and are often more seriously affected than familial cases [3]. Gene mutations in *FBN1* have been demonstrated in 92% of classically affected MFS type 1 cases [4,5]. Other genes capable of causing familial ascending thoracic aortic aneurysms are now being described, but these families can usually be differentiated clinically from MFS [6-9].

Aneurysmal dilatation of the aortic root is the most serious cardiovascular manifestation of MFS. This results from weakening of the tissues within the aortic wall and consequent reduced ability to contain the forces associated with cardiac ejection. The natural history of aortic root aneurysms is expansion over many years followed by dissection and rupture and premature death. In addition, myxomatous valve changes with insufficiency of the mitral and aortic valves, and progressive myocardial dysfunction can also occur and require intervention, and a wide range of non-cardiac manifestations affecting skeletal and ocular systems result in significant morbidity and mortality. The average age at death of an untreated MFS patient is 32 years [10].

Current treatment includes careful follow-up and prophylactic surgical intervention to replace the aneurysmal root when the risk of spontaneous dissection or rupture exceeds that of surgery. For most patients this risk is judged by the size of the aortic root, with the standard threshold at which patients are usually considered for prophylactic surgical treatment being 50 mm [11]. In some patients with an adverse family history, or where pregnancy is considered or where rapid dilatation is observed, surgical intervention may be considered at diameters below 50 mm.

### Medical treatment

The goal of medical therapy in MFS is to slow or arrest the development of clinical manifestations of MFS. With respect to the cardiovascular system, the current gold standard for medical treatment is with oral beta blockers. Beta blocker therapy has been shown in retrospective and prospective studies to reduce the rate of aortic root dilatation [12-15], and is associated with an increase in life span [16]. The mechanism is unknown but is likely to be mediated through reduction in left ventricular ejection force, blood pressure, and pulse pressure, all of which potentially reduce aortic wall stress. However, recent studies in children with MFS and a meta-analysis have cast doubt on the efficacy of beta blocker therapy [17]. Furthermore, many patients cannot tolerate beta blockers (approximately 25% to 50% of MFS patients), either because they have asthma, which affects about 20% of MFS children, or because of intolerable side effects including dizziness, nightmares, and lethargy, or can only tolerate them in small doses. Furthermore, beta blocker therapy does not alter the underlying process that results in weakness and dilatation of the aortic wall.

### Fibrillin-1

Fibrillin-1 is the major component of extracellular myofibrils which form the backbone of the elastic tissues in the extracellular matrix. Original hypotheses of the pathogenesis of MFS were based on a simple model of aortic dilatation occurring as a mechanical consequence of abnormal elastic tissues. However, such a hypothesis does not explain many manifestations of MFS including excessive growth and abnormal alveolar septation.

Elucidating the mechanisms of aortic dilatation has been facilitated by the development of an MFS knockout mouse. The so-called mgR mouse has an identical mutation of fibrillin-1 as that seen in human MFS and the mutant allele produces structurally normal fibrillin-1 protein at 15% of the normal level. The mouse manifests all the clinical features of human MFS including the mouse equivalent of postnatally-acquired aortic disease and death by aortic dissection, as well as lung and skeletal findings [18,19]. Homozygous mgR mice die between 3 and 6 months of age of dissecting aortic aneurysm. The mice show loss of structural integrity of the aortic wall with cystic medial necrosis, histologically identical to that seen in human MFS. Breach of the elastic laminae in fibrillin-1 mutant mice is believed to allow infiltration of inflammatory cells into the media, resulting in intense elastolysis associated with increased expression of matrix metalloproteinases (MMPs) [20].

Research in this mouse model has elucidated a much more complex role for fibrillin-1 in the regulation of extracellular activity of transforming growth factor beta (TGF-β). Abnormal fibrillin-1 leads to excess activity of TGF-β in extracellular tissues and this appears to contribute to the pathogenesis of many of the phenotypic features of MFS [21]. Myxomatous changes of the mitral valve in mutant mice were correlated with excess TGF-β signaling, and prevented by TGF-β antagonism *in vivo*[22]. Furthermore, increased TGF-β signaling in association with increased MMP expression was also observed in the dura and aortic wall of fibrillin-1-deficient mice [23]. These mice were shown to have excess immunoreactive free TGF-β, and systemic administration of a TGF-β neutralizing antibody rescued lung morphogenesis in fibrillin-1-deficient mice, and attenuated changes in the aortic wall.

### The renin-angiotensin system and TGF-β regulation

Extracellular TGF-β is also regulated by the autocrine molecule angiotensin II. Activation of the angiotensin II receptor type 1 (AT1) can increase the production of TGF-β, which may be responsible for many of the cellular events in the tissue of patients with MFS including proliferation of vascular smooth muscle cells and levels of MMPs. By contrast, activation of the angiotensin II receptor type 2 (AT2) has beneficial effects on aortic wall homeostasis. Selective inhibition of the AT1 receptor therefore offers a therapeutic target to favorably modify the pathogenesis of tissue injury in MFS. AT1 receptor blockers (ARBs) include a number of commonly used antihypertensive medications including losartan and irbesartan. In the experimental mouse, ARB administration resulted in a clinically relevant decrease in TGF-β signaling, reduced plasma levels of free TGF-β, reduced tissue expression of TGF-β-responsive genes, and reduced levels of intracellular mediators within the TGF-β signaling cascade, such as phosphorylated Smad2.

Habashi *et al*. [24] reported that five young Marfan mice were given 0.6 g/L of losartan, consumed through their drinking water for a period of 6 to 10 months. Another group of ten MFS mice were given a placebo, and a third group of seven MFS mice were given a dose of 0.5 g/L of propranolol, a beta blocker. A fourth group of eleven wild-type mice without MFS served as a control group. The mice studied were 2 months old when therapy was started, equivalent to human teenage years. These mice already had enlarged aortas. After the mice were treated for 6 months, examination of the aorta histologically showed losartan, as opposed to placebo or propranolol, prevented elastic fiber fragmentation, and blunted TGF-β signaling in the aortic media. Additionally, echocardiographic measurements of aortic root growth in losartan MFS mice were comparable to the normal control group of mice (*P* = 0.55), and the absolute aortic root diameter between losartan MFS mice and the normal control group at the end of treatment was also similar (*P* = 0.32). Losartan MFS mice also showed significant improvement in aortic wall thickness and aortic wall architecture compared to placebo, and normalization relative to the normal control group. In comparison, propranolol-treated MFS mice showed a slower rate of aortic growth compared to the placebo group (*P* <0.001), but showed no effect on aortic wall thickness or aortic wall architecture compared to the placebo group, thus limiting its effect in slowing the rate of aortic growth.

Therefore it is particularly attractive to consider the use of an ARB, which both lowers blood pressure comparably with beta blocker therapy [25-28] (a known positive effect in MFS) and leads to a clinically relevant decrease of TGF-β signaling [29,30]. These data support the hypothesis that many features of MFS are probably due to failure of proper regulation of TGF-β function [31,32].

### ARBs in human MFS

Pilot data of 18 severe neonatal MFS cases [33,34] treated with losartan [31] indicated that placing an affected infant on an ARB was associated with a reduction in the rate of aortic root dilatation. Preliminary data indicated the average change in aortic root diameter pre-losartan treatment was 3.5 mm/yr and following losartan treatment was 0.5 mm/yr. This compares with an average change in aortic root diameter of 1.67 mm/yr in patients treated with beta blockers alone. This pilot data suggests that a clinical trial of an ARB may demonstrate effects more specific than, and probably additive to, beta blockers [35-37].

In the Aortic Irbesartan Marfan Study (AIMS), we wish to translate the pre-clinical, neonatal, and pediatric work into a randomized clinical trial (RCT) to investigate whether the ARB irbesartan can reduce aortic dilatation in patients with MFS compared to placebo. All patients will receive standard treatments including beta blocker therapy if tolerated. We believe this will provide additional information regarding the effects of combined therapy as well as the effects in patients intolerant of beta blockers. Furthermore, we propose to examine the effects of irbesartan in a wider age range, extending the upper adult age limit to 40 years, since patients are often first diagnosed in adulthood.

A study funded by the National Institute of Health (NIH) of losartan versus beta blocker is being carried out in the USA. This study has recruited 604 patients and is also evaluating effects on aortic dilatation [38]. The USA study has a different design to the one described in this protocol, since the AIMS trial is comparing irbesartan versus placebo, but the two trials will provide complementary information on this important question.

It is widely accepted that aortic root dilatation is the hallmark of serious cardiovascular complications in MFS. Furthermore, aortic root dilatation is usually the major factor considered in referring patients for surgery. Therefore, while being a surrogate outcome measure, clinical outcome and the decision to intervene are directly related to aortic dilatation. We have considered conducting a clinical outcome study in MFS (evaluating the effects of irbesartan on rates of death, aortic surgery, or other serious cardiovascular complications) but this study would probably take 10 years or more to complete in order to assess the true long-term effects of treatment. For this reason we have selected the rate of aortic root dilatation as the primary outcome measure. We believe that the main benefit of ARB treatment will be in the prevention of aortic complications when applied as a prophylactic measure in patients with MFS who are either in their growing years or in adulthood prior to the development of severe dilatation. We consider that this is too long to wait to introduce a potentially lifesaving treatment for young patients at risk of severe complications. It is important to be aware of the level of evidence needed to introduce a new treatment for MFS, but the carefully considered opinion of the MFS experts collaborating in this study is that a significant reduction in aortic dilatation would provide important clinical benefits to patients, and that this measure is a robust surrogate for clinical outcomes. Previous studies that showed the benefits of beta blockers in MFS used the same outcome of aortic dilatation as the primary outcome measure. We also know that there is increasing use of ARBs among patients with MFS even though there is no clear evidence of efficacy or indeed safety for this indication, and thus there is an urgent need to complete studies of the efficacy of ARBs in MFS.

### Feasibility

MFS patients require lifelong monitoring and many need major surgery. As a healthcare burden comparison there are about 8,000 patients with Cystic Fibrosis in the UK who generally require a greater level of ongoing healthcare support than MFS patients, but there are important similarities in that both conditions are inherited, affect young people, and reduce life expectancy.

MFS patients currently suffer great fear about their long-term prognosis and if effective, irbesartan would provide a lifesaving treatment option which could extend life span into the normal range. There is tremendous support from the medical community and lay MFS population to perform this trial.

### Aim of study

To investigate whether the angiotensin II receptor antagonist irbesartan reduces the rate of aortic dilatation in MFS compared to placebo.

## Methods/Design

This trial is an investigator-led, prospective, randomized, placebo-controlled, double-blind, phase III, multicenter study. Patients will be randomized to two groups:

1. Irbesartan group

2. Placebo group.

### Patient population

#### Inclusion criteria

1. Clinically confirmed MFS using the revised Ghent diagnostic criteria (2010)

2. Provision of informed consent

3. From ≥6 to ≤40 years of age inclusive.

#### Exclusion criteria

1. Previous cardiac or aortic surgery

2. Planned cardiac or aortic surgery at the time of randomization

3. Aortic root Z-score ≤0

4. Aortic diameter ≥4.5 cm

5. Hemodynamically significant, severe valvular disease (at the judgement of the treating clinician)

6. Heart failure, defined as left ventricular ejection fraction <40%

7. Therapeutic use of angiotensin-converting-enzyme (ACE) inhibitors/angiotensin II receptor antagonist (patients on ACE inhibitors/angiotensin II receptor antagonists who discontinue this treatment are required to have a 3-month wash-out period prior to entry)

8. Previous recorded adverse reaction to the trial medication (irbesartan) or any ACE inhibitor/angiotensin II receptor antagonist

9. Female patients who are pregnant, planning pregnancy, or not using reliable contraception

10. Known impaired renal function defined as estimated creatinine clearance <60 mL/min, or serum creatinine >150 μmol/L.

### Concomitant treatments and procedures

Patients should generally continue all their concomitant routinely indicated treatments including beta blockers. Beta blocker use is not mandated by this protocol and should be used at the judgement of the treating physician.

Therapeutic use of ACE inhibitors or other angiotensin II receptor antagonists during the trial is not permitted. Patients are eligible for the trial if they have a 3-month wash-out period (no ACE inhibitors/angiotensin II receptor antagonists) prior to entry.

### Screening, randomization, and unblinding

#### Screening

Potentially eligible patients will be screened at participating centers throughout the UK. All patients diagnosed clinically as having MFS within the participating hospital clinics will be screened for eligibility. Those who are identified as potentially suitable will be approached to see if they wish to participate. Written informed consent will be requested before the patient is enrolled into the study. Family members who are affected can also be screened. Patients will be jointly supervised by the responsible cardiologist and geneticist at each site.

#### Randomization

Randomization will be carried out by an internet-based randomization service. Investigators will be required to confirm that the patient is eligible. Patients will be stratified at randomization according to age (6 to 18 years and >18 years of age), beta blocker use, and center.

#### Unblinding

Unblinding the allocation code can only be undertaken in exceptional circumstances via the electronic case record form (eCRF), when knowledge of the allocation is essential for treating the patient, for example, suspected unexpected serious adverse reaction (SUSAR) or other serious adverse event (SAE). The Clinical Trials and Evaluation Unit (CTEU) at the Royal Brompton and Harefield NHS Foundation Trust, London, UK, will be contacted before breaking the code. In all cases, the date and reason for breaking the code will be documented. Unblinding will occur at the individual patient-level only.

### Trial intervention

Study treatment will be in three phases:

1. Run-in phase: 75 mg once daily (OD) open-label irbesartan for 4 weeks before randomization

2. Treatment phase: 150 mg (OD) active/placebo for 4 weeks before uptitration in appropriate patients

3. Treatment phase: 300 mg (OD) active/placebo maximum tolerated dose for remaining treatment period.

The proposed target doses are as follows: 300 mg OD for patients >50 kg and 150 mg OD for patients ≤50 kg. Patients will be monitored at regular intervals in the baseline phase for tolerability to study medication including general clinical examination, blood pressure, electrolytes, and renal function. Compliance and tolerability will also be monitored by the study teams at 3-month intervals by telephone, and should there be any issues the patient will return to clinic for review. Indications for stopping the study drug would include any apparent serious side effects, hypotension not amenable to a reduction in study drug, pregnancy, or significant impairment of renal function.

### Study visits

#### Visit 0, run-in phase (month 1)

Eligible patients screened from outpatient clinics will be invited to consent to the study. If the patient consents, they will undergo a clinical examination including blood pressure check and electrocardiography (ECG). Patients will be dispensed with a 1-month supply of 75 mg open-label irbesartan. This is to establish tolerance and compliance to irbesartan prior to the baseline study visit. Patients will also undergo the baseline echocardiogram and have study-specific bloods taken for renal function, mutation analysis (if not already taken as part of routine clinical care), and TGF-β sub-study.

#### Visit 1, baseline (month 2)

Eligible patients who tolerate the open-label run-in phase will have a clinical examination and baseline characteristics recorded (height, weight, blood pressure, heart rate). Patients will also undergo a compliance to medication check, clinical evaluation including ECG, medications review, liver function tests, full blood count, urea and electrolytes, and renal function. Baseline medication will remain unchanged (beta blocker, other antihypertensive, or nil).

Patients will then be randomized into the trial using the interactive voice recognition system (IVRS). A unique identification number will be allocated to the patient, which will match the number on the study drug held at the site pharmacy. Once allocated, the study drug will be dispensed by the pharmacy. Patients will be provided with the 150 mg dose of irbesartan/placebo for 1 month. Children ≤50 kg will continue with the 150 mg dose and will not be uptitrated.

#### Visit 2, uptitration or maintenance visit (month 3)

Patients >50 kg will be uptitrated to the 300 mg dose of irbesartan/placebo at month 3 if tolerated. Patients will undergo a compliance to medications check, clinical evaluation including blood pressure check, medications review, liver function tests, full blood count, urea and electrolytes, and renal function tests.

Patients who remain on the 150 mg dose will undertake the visit procedures described above, although they will not be dispensed the 300 mg dose.

#### Visit *, titration visit (if necessary)

Patients who do not tolerate the maximum 300 mg dose for whatever reason will be downtitrated to the 150 mg dose and continue in the trial. At this visit, the patients will have a clinical evaluation including blood pressure, medications review, liver function tests, full blood count, urea and electrolytes, and renal function tests, before the 150 mg dose is dispensed.

### Telephone checks (3-month intervals)

Subsequent to visit 2, there will be 3-month interval telephone calls between the research team and patient to check compliance to the medication and tolerability up to month 60 (5 years). Should the patient have any problems they will return to the clinic for further review by the research team.

### Annual visits (month 12, 24, 36, 48, and 60)

Patients will also have an annual follow-up at month 12, 24, 36, 48, and 60 (depending on entry to the trial) as per routine clinical care to undergo a compliance to medication check, clinical evaluation including blood pressure, ECG, medications review, liver function tests, full blood count, urea and electrolytes, renal function, and drug dispensing. Patients will also undergo an annual echocardiogram for analysis. At year 1, an annual study-specific blood sample will be taken for analysis for the TGF-β sub-study.

Study procedures and follow-up are described in Table 1.

**Table 1:** Study-related investigations and follow-up

**Procedure**	**Visit 0**	**Visit 1**	**Visit 2**	Visit *	**Telephone call**	**Visit 3**	**Telephone call**	**Visit 4**	**Telephone call**	**Visit 5**	**Telephone call**	**Visit 6**	**Telephone call**	**Visit 7**
	**Month 1, run-in phase, 75 mg open-label**	**Month 2, baseline, 150 mg active/placebo for 1 month**	**Month 3, uptitration to 300 mg active/placebo or maintenance at 150 mg active/placebo**	**Month 4, titration visit (if necessary)**	**Month 6, 9**	**Year 1 (month 12)**	**Month 15, 18, 21**	**Year 2 (month 24)**	**Month 27, 30, 33**	**Year 3, (month 36)**	**Month 39, 42, 45**	**Year 4 (month 48)**	**Month 51, 54, 57**	**Year 5 (month 60)**
Screening	√													
Compliance check			√	√		√	√	√	√	√	√	√	√	√
Clinical evaluation	√	√	√	√		√		√		√		√		√
Informed consent	√	√												
Randomization		√												
Blood pressure	√	√	√	√		√		√		√		√		√
Echocardiogram	√					√		√		√		√		√
ECG	√	√				√		√		√		√		√
Medications	√	√	√	√		√		√		√		√		√
Liver function		√	√	√		√		√		√		√		√
Full blood count		√	√	√		√		√		√		√		√
Urea and electrolytes		√	√	√		√		√		√		√		√
Renal function	√	√	√	√		√		√		√		√		√
Study drug given	√	√	√	√		√		√		√		√		
Mutation analysis (if necessary)	√													
Blood sample (TGF-β and other biomarkers)	√					√								

Visit * only required if patient does not tolerate 300 mg dose. Patient will be downtitrated to 150 mg OD. ECG, electrocardiography; OD, once daily; TGF-β, transforming growth factor beta.

### Participant follow-up

Patients will also be followed up via the NHS Information Centre Medical Research Information Service for a minimum of 20 years after the end of study follow-up.

### Pregnancy

Women of childbearing potential (able to have children) are required to use reliable forms of contraception if taking part in the study (including barrier and/or oral methods). Women of childbearing potential will be excluded if planning pregnancy during the trial period or sexually active and not using a reliable form of contraception.

### Patient withdrawal

#### Temporary discontinuation of investigational medicinal product (IMP)

There should be no reason to discontinue the study drug unless in exceptional circumstances such as side effects, for example renal impairment or significant hypotension. In such cases, the investigator should downtitrate or discontinue the study medication as required. Reintroduction of the study medication should be monitored and uptitration undertaken as required.

#### Permanent discontinuation of IMP

Patients may permanently withdraw from treatment with the investigational medicinal product (IMP) if they decide to do so, at any time and for any reason, or this may be the investigator’s decision. If there is considered to be a concern about the batch of IMP, recall procedures will be in place and measures will be taken to ensure the safety of all trial participants.

### Withdrawal from trial procedures and incomplete follow-up

Patients are free to withdraw consent from trial procedures at any time. Investigators must ascertain the reasons for the withdrawal including discontinuation of study drug, withdrawal from study investigations and/or follow-up, withdrawal due to adverse events, failure to attend, non-compliance, withdrawal of consent, or other reasons. The withdrawal form must be faxed to the CTEU within 5 working days, unless withdrawal is due to a SAE, in which case the investigator will follow SAE reporting procedures.

Withdrawal from trial procedures may result in incomplete patient follow-up and failure to capture outcome data. In these cases as much data as possible will be collected, up until the point of withdrawal. Patients may choose to withdraw from trial procedures and request that further data are not collected.

### Enrolment and participating centers

Based on 26 centers recruiting, we expect an approximate recruitment rate of one patient/month/center, which will enable 490 patients to be recruited over a 2-year enrolment window. The study will run for 66 months (5.5 years) split into four periods as follows:

1. Start-up: 6 months

2. Enrolment: 24 months (with potential to extend into the third year if necessary)

3. Follow-up: 36 months minimum (patients enrolled at the start of the study will be followed up for a maximum of 60 months, thus the mean follow-up period is 48 months)

4. Closeout: 6 months.

### Outcome measures

#### Primary outcome measure

The primary outcome measure will be the absolute change in aortic root diameter per year measured by echocardiography. Echo measurements will be taken by transthoracic echocardiograms (TTEs) performed at baseline and annually thereafter in order to assess the annual change of aortic dilatation.

#### Secondary outcome measures

The secondary outcome measures are as follows:

1. Change in Z-score per year, where the Z-score is calculated on aortic root and body surface area (BSA)

2. Clinical events and requirement for surgery including aortic dissection confirmed by transesophageal echocardiography (TEE), MRI, or CT, aortic dissection requiring emergency surgery, aortic dissection requiring elective surgery, aortic dilatation requiring elective or emergency surgery, death (all causes and classified by suspected cause), cerebrovascular accident, cardiovascular death, aortic regurgitation requiring surgery, or death during surgery for any of the above

3. Left ventricular function determined by volumes and ejection fraction

4. Left ventricular mass measurements

5. Assessment of valvular function

6. Cardiac rhythm and voltage

7. Height, weight, arm span, and lower segment measurements

8. Fibrillin-1 mutation analysis will be performed for those patients whose mutation status is unknown.

### Echocardiography

Aortic dilatation will be measured by echocardiography. All annual echocardiograms will be analyzed by an established echo core laboratory. Patients will be seen in centers that have routine access to standard M-mode, two-dimensional (2-D) echocardiography. Transthoracic M-mode, 2-D, and Doppler echocardiograms will be performed by experienced technicians, according to a standardized protocol. Each center will be trained on the standardized protocol prior to commencing the trial. Aortic root diameter will be measured at the annulus, in the sinuses of Valsalva at the tip of the open cusps at 90° to the direction of flow, the sinotubular junction, ascending aorta, aortic arch, and descending aorta.

Each echocardiogram will be sent electronically to the echocardiographic core laboratory at the John Radcliffe Hospital, Oxford, UK, where a single experienced investigator will supervise the reading and interpretation of all echocardiograms according to a standardized protocol to reduce variability due to observer variation. The echo core laboratory will be blinded to the study drug allocation to eliminate any bias in echo measurement. Quality control processes will be developed as part of the echo protocol. The echocardiographic protocol is included in Additional file 1.

### Fibrillin-1 mutation analysis at baseline

There is a 92% chance of finding a mutation in classical MFS. For patients who have not already had fibrillin-1 mutation screening, but who are considered clinically affected with MFS, a 5 mL EDTA blood sample will be collected and stored for assay. Samples will be analyzed and funded as per usual local arrangements.

Samples from units which do not have funding for these tests should be sent directly to the Sonalee Laboratory, St. George’s, University of London, London, UK [3]. Samples will be entered in the research program assay, and research laboratory reports will be issued.

A separate genetic sub-study will be undertaken to investigate the correlation between the response to medication and the site and type of mutation which determine the phenotype [4,39,40]. Patients will consent to this sub-study separately from the main trial.

### Additional investigations and sub-studies

To increase knowledge about the underlying disease and the potential mechanism of action of ARBs, several sub-studies are proposed. The main proposed sub-studies are:

1. Genetic sub-studies: a) fibrillin-1 mutation analysis; and b) pharmacogenetics sub-study

2. Total circulating TGF-β1.

### Data collection

#### Electronic case record form (eCRF)

Trial data will be captured on a web-based eCRF. The eCRF will be designed in accordance with the requirements of the trial protocol and will comply with regulatory requirements. Access to the eCRF will be password-protected.

#### Pharmacovigilance

The Royal Brompton and Harefield NHS Foundation Trust (Sponsor) has delegated responsibility for pharmacovigilance to the trial coordinating center, the CTEU of the Royal Brompton and Harefield NHS Foundation Trust. The CTEU will be responsible for recording all reported SAEs from investigational trial sites, and expedited reporting of SUSARs in accordance with statutory regulations.

#### Adverse event (AE)

An adverse event (AE) is defined as any untoward medical occurrence in a patient or clinical trial subject administered a medicinal product and which does not necessarily have a causal relationship with this treatment. An AE can therefore be any unfavorable and unintended sign (including an abnormal laboratory finding), symptom, or disease temporally associated with the use of an IMP, whether or not considered related to the IMP.

#### Adverse reaction (AR)

Adverse reactions (ARs) are all untoward and unintended responses to an IMP related to any dose administered. All AEs judged by either the reporting investigator or the sponsor as having reasonable causal relationship to a medicinal product qualify as ARs.

In the event an AR is reported during the trial, investigators will assess the severity of the AE using the following criteria, detailed on the AE report form in the eCRF:

1. Mild: awareness of signs or symptoms, but easily tolerated; are of minor irritant type; causing no loss of time from normal activities; symptoms would not require medication

2. Moderate: discomfort severe enough to cause interference with usual activities

3. Severe: inability to do work or usual activities; signs and symptoms may be of systemic nature or require medical evaluation and/or treatment.

### Unexpected adverse reaction (UAR)

An unexpected adverse reaction (UAR) is an AR, the nature or severity of which is not consistent with the applicable product information (summary of product characteristics, SmPC). When the outcome of the AR is not consistent with the applicable product information this AR should be considered as unexpected. Side effects documented in the SmPC which occur in a more severe form than anticipated are also considered to be unexpected.

### Expected drug-related AR

Expected drug-related ARs will be referred to the SmPC as provided and the summary in Additional file. Symptomatic hypotension defined as systolic blood pressure <90 mmHg in combination with dizziness and/or syncope on standing is considered an expected AR and will be reported on a specific AE form. Any other AR will be reported on a generic AE form on the eCRF.

### Reporting ARs

Investigators will report all ARs on the AE report form in the eCRF including information of the event, details of date of onset, frequency, severity, and potential relationship to treatment, outcomes, and action taken. Investigators will make a clinical judgement as to the appropriate action required depending on the severity of the reaction. This could include monitoring the patient over a period of time, interrupting the drug regime, discontinuing the patient from the trial, or continuing with the trial as specified. Investigators will submit AE reports to the CTEU after each patient visit. The CTEU will maintain a database of all ARs. AEs will be reviewed at regular intervals by the Data Monitoring Committee (DMC) for signal and trend analysis.

### Serious adverse events (SAEs)/reactions

SAEs or reactions are defined as any untoward medical occurrence or effect that at any dose results in death, is life threatening, requires hospitalization or prolongation of existing inpatient hospitalization, results in persistent or significant disability or incapacity, or is a congenital anomaly or birth defect.

Should a study participant become pregnant while undertaking the trial, or aid in the conception of a child while they are participating in the trial, the pregnancy and resulting child should be followed up for a period of no less than 18 months. In this trial should a child be followed up and diagnosed with MFS, this would not be considered unexpected due to the nature of the syndrome.

### Expected SAEs (as a result of the underlying disease)

Expected SAEs as a result of the underlying disease include: admission or procedure for MFS including treatment for cardiovascular, musculoskeletal, ocular, and thoracic complications; aortic dissection; aortic regurgitation requiring surgery; emergency or elective aortic root and/or valve replacement surgery; cerebrovascular accident; and cardiovascular death, sudden death, or death during surgery.

Expected SAEs will be reported as per the usual data capture requirements for the study and are not subject to expedited reporting.

Other SAEs which are not expected irrespective of causality will be subject to SAE reporting requirements. In the event of an SAE, investigators will report details on the SAE form on the eCRF including date of event, admissions, diagnosis details, date of discharge, or death. SAE reports must be completed within 24 hours of the investigator’s knowledge of the SAE. Investigators will be able to submit follow-up SAE reports should further information become available. Investigators will be expected to assess and assign causality and expectedness of each event on the form using the definitions described below. The CTEU will review all SAE reports. The Chief Investigator/deputy will review the SAE reports and inform the CTEU of the assessment.

### Definitions for assessment of causality

The definitions for assessment of causality include:

1. Unrelated: there is no evidence of any causal relationship

2. Unlikely: there is little evidence to suggest there is a causal relationship (for example the event did not occur within a reasonable time after administration of the trial medication). There is another reasonable explanation for the event (for example the patient’s clinical condition, other concomitant treatment)

3. Possible: there is some evidence to suggest a causal relationship (for example because the event occurs within a reasonable time after administration of the trial medication). However, the influence of other factors may have contributed to the event (for example the patient’s clinical condition, other concomitant treatments)

4. Probable: there is evidence to suggest a causal relationship and the influence of other factors is unlikely

5. Definitely: there is clear evidence to suggest a causal relationship and other possible contributing factors can be ruled out

6. Not assessable: there is insufficient or incomplete evidence to make a clinical judgement of the causal relationship.

### Suspected unexpected serious adverse reactions (SUSARs)

A serious adverse reaction (SAR) can be considered unexpected when the AR is not consistent with the applicable product information or expected SAEs listed above. All SUSARs related to an IMP, which occur during the trial, are subject to expedited reporting. Where applicable, if an event is considered a SUSAR, the patient should be unblinded from the study allocation.

### Reporting of SUSARs

A full and detailed account of the SAE must be recorded on the SAE report. The SAE report must be completed within 24 hours. A medical summary should also be faxed to the CTEU within 24 hours. The Chief Investigator/deputy will review the report and summary and inform the CTEU of the assessment.

### Expedited reporting of SUSARs

All SUSARs will be reported to the Medicines and Healthcare Products Regulatory Agency (MHRA) and NHS Research Ethics Committee (REC) by the CTEU. All SUSAR reports will be unblinded prior to submission. A SUSAR which is fatal or life threatening will be reported to the MHRA and the main REC by the CTEU as soon as possible and within 7 days of knowledge of the event. A SUSAR which is not fatal or life threatening must be reported to the MHRA and the main REC as soon as possible and within 15 days of knowledge of the event. The CTEU will inform all relevant parties of any reported SUSARs within 15 working days.

### Annual reporting

The CTEU will submit annual safety reports of all suspected SARs in accordance with regulatory requirements to the MHRA and the main REC. Annual safety reports will be submitted to the MHRA on the date of the original clinical trials authorization. Annual progress reports will also be submitted to the main REC. There is no requirement for local trial sites to submit progress reports to local RECs.

### Statistical considerations

#### Sample size calculation

The primary outcome is absolute change in aortic root diameter per year. The sample size calculation was based on estimates obtained from a database of MFS patients maintained by AC, co-investigator and lead Geneticist. Information was extracted from this database for all patients who met the following criteria: age at first echo between 6 and 40 years; at least two serial echo measurements; and time between the first and last valid measurement between 0.9 and 5.1 years.

This provided a database of 254 patients who had a median (interquartile range) follow-up time of 3.4 (2.4, 4.5) years. The data were cleaned and reviewed and during this time the average rate of aortic dilatation was approximately 1 mm per year, with a standard deviation of 1.8 mm. Table 2 shows the number of patients required to test for a difference between an annual dilatation rate of 1 mm on placebo against hypothesized rates on irbesartan. This is based on achieving 80% power and testing at the 5% significance level.

**Table 2:** Estimated annual dilatation rates over a mean follow-up of 3.4 years

**Number of patients in placebo group**	**Number of patients in irbesartan group**	**Annual growth on placebo (mm)**	**Annual growth on irbesartan (mm)**	**SD of annual growth (mm)**
142	142	1	0.4	1.8
169	169	1	0.45	1.8
204	204	1	0.5	1.8
252	252	1	0.55	1.8
318	318	1	0.6	1.8

By inspection of this table, to detect a 0.5 mm change in dilatation rate would require 204 patients in each group. Allowing for a 20% drop-out (including missing data and non-compliance) we aim to recruit 245 patients in each group, making a total of 490 patients. Expansion rates according to treatment group would be regularly reviewed by the independent Data and Safety Monitoring Committee (DSMC) who would advise the Steering Committee if there was clear evidence of benefit before the scheduled end of the study, or alternatively may advise if there is no real possibility of finding a difference (performing a 'futility’ analysis) either because irbesartan does not have the expected effects, or if aortic expansion rates are slower than expected in the control group. We have selected a proportional reduction of 50% of dilatation rate per annum (0.5 mm) largely on pragmatic grounds. If irbesartan only offered a very modest reduction in rate we are much less certain that this could be translated into a clinical benefit. We are also measuring a surrogate outcome (aortic dilatation) but one which is closely related to adverse clinical events in this population.

### Statistical analysis

The primary analysis in this study is the comparison of the rate of annual aortic dilatation in those patients treated with irbesartan compared to placebo. The annual rate of dilatation will be calculated by estimates of mean values of the annual echocardiograms adjusting the time of follow-up to a 'common’ start point, that is, the baseline measurement. An independent samples *t*-test will be used to test for a difference in the rate between the irbesartan and placebo groups, assuming that the measurement of annual dilation follows a normal distribution. Analysis of covariance (ANCOVA) will be used if the analysis of the primary outcome is adjusted for any other variables. The primary analysis will be carried out according to the intention-to-treat principle in which all randomized patients will be included according to their initial randomized allocation irrespective of whether they continue to take the assigned treatment or not. Patients who are not followed up to the end of the study for whatever reason will have the last echo measurement included in the analysis. Patients will be followed from the time of randomization to the end of the study period. Thus individual patients will have a variable follow-up period. No formal method for imputing missing aortic diameter values is proposed as the comparison will be performed on the mean of group data. Secondary analyses include comparison of other measurements using appropriate comparative and descriptive statistics.

### Regulatory and ethical considerations

#### Regulatory framework and approval

This study is a randomized trial of an IMP (licensed product in new conditions of use; new dosing schemes/new target population), and as such will comply with the European Clinical Trials Directive and the Medicines for Human Use (Clinical Trials) Regulations 2004. A clinical trial authorisation (CTA) has been granted from the MHRA. The study is registered in the European Clinical Trials Database with a European Union Drug Regulating Authorities Clinical Trials (EudraCT) number.

#### Ethical approval

The trial will comply with the Declaration of Helsinki (http://www.wma.net/) on research involving human subjects. The study protocol, patient information sheet, and consent form have been approved by the NHS National Research Ethics Service (NRES) and subsequently the research and development departments of each participating center for site-specific approval. The AIMS trial is also approved on the National Institute for Health Research (NIHR) portfolio.

### Monitoring

#### Initiation visit

Before the study commences, each trial site will receive a training visit from the CTEU where required. The purpose of these visits will be to ensure that the local research team (local principal investigator, co-investigators, study coordinator, and pharmacists) fully understand the protocol, eCRF, and the practical procedures for the study.

### Interim monitoring visits

At regular intervals during the study, the CTEU will perform monitoring visits to each trial site. The purpose of these visits is to ensure compliance with the protocol and that ethical and regulatory requirements are met. Source data verification (SDV) and checking of essential documents will be performed. Monitors will also visit the pharmacy departments to review study procedures, storage, and accountability of the IMP.

Monitoring visits also provide an opportunity for further training if required (for example new staff). Central review of study data will also be performed throughout the study by the data management team at the CTEU.

#### Closeout visit

At the end of the study, each trial site will receive a closeout visit from the CTEU to resolve any outstanding edit queries or AEs and to verify the archiving procedures for study documentation.

### Trial organization and committees

#### Study management

The study will be sponsored by the Royal Brompton and Harefield NHS Foundation Trust. The Chief Investigator will be MM at the Royal Brompton Hospital, London, UK. AC at St George’s Hospital will be the lead Geneticist and XYJ at the John Radcliffe Hospital will be the lead Echocardiologist for the study. All participating clinicians have extensive experience of running MFS diagnostic clinics. A Trial Steering Committee (TSC), DMC, and Trial Management Group will be convened to oversee the trial. Central coordination of this clinical trial will be provided by the CTEU.

### Trial Steering Committee (TSC)

The main role of the TSC is to monitor and supervise the progress of the trial. The composition of the TSC will comply with Medical Research Council (MRC) guidelines with an independent Chair and lay representation as well as the Chief Investigator and main co-investigators. The TSC will meet regularly throughout the study.

### Data Monitoring Committee (DMC)

All members of the DMC are independent of the trial. The DMC will meet prior to the start of the trial and then one and two thirds of the way through the trial, or as required thereafter. The DMC will be expected to develop, in agreement with the investigators, a charter outlining their responsibilities and operational details.

### Study coordination

The study will be coordinated and managed by the CTEU, a dedicated clinical trials department within the Royal Brompton Hospital. In addition to providing overall project coordination, the CTEU will assist in preparing the final protocol, the investigators’ manuals, design the eCRF, provide the randomization service and design, and instigate the data management system. The CTEU will ensure that the trial runs according to the pre-agreed timetable, recruitment targets are met, eCRFs are completed accurately, compliance with relevant ethical and regulatory standards, and that all aspects of the study are performed to the highest quality. The CTEU will also assist in the training of investigators and coordinators at the start-up of the study and for performing monitoring and pharmacovigilance procedures throughout.

### Investigators’ responsibilities

Investigators are required to ensure compliance to the protocol and all statutory regulations and guidelines, eCRFs, and manual of operations. Investigators are required to allow access to study documentation or source data on request for monitoring visits and audits performed by the CTEU, sponsor, or any regulatory authorities.

### End of trial

The end of trial will be declared when the last patient recruited completes the last follow-up visit, that is, echocardiogram at 36 months follow-up visit.

### Investigational medicinal products

#### Manufacture

The study drug (irbesartan and placebo) will be purchased from the commercial supplier, Sanofi-Aventis, Guildford, Surrey, UK, which holds the manufacturing license to produce the IMP.

Brecon Pharmaceuticals Ltd, Hay-on-Wye, UK, will undertake to prepare, pack, and label the IMPs and distribute as required throughout the trial. The IMP supply will be labeled in accordance with regulatory requirements and specifications and will be approved by the MHRA as part of the application for CTA.

### Storage and dispensing

The study drug patient kit for the 1-month run-in phase, baseline phase, and uptitration phase (month 1 to 3) will be stored in a secure area of the pharmacy, under the conditions described in the respective SmPC.

The IMP supply will be dispensed by the local pharmacy which will be responsible for maintaining a record of accountability.

Continuation packs of the study drug will be dispensed direct to patient homes on a 3-month supply basis by Brecon Pharmaceuticals Ltd. Drug accountability records will be supplied as required.

### Publication policy and dissemination of results

The results from the trial will be submitted for publication in a major journal irrespective of the outcome. The TSC will be responsible for approval of all manuscripts arising from the study prior to submission for publication. Sub-studies of center-specific data may only be carried out with the knowledge and approval of the TSC. Sub-study publications must not be published prior to the publication of the main study.

All publications and presentations will make appropriate acknowledgement of the contribution of the collaborative group. At the end of the study, patients will be able to request a copy of the results of the study from the investigator at that site.

## Trial status

The first patient was enrolled in March 2012, and recruitment is ongoing.

## Abbreviations

2-D: Two-dimensional; ACE: Angiotensin-converting-enzyme; AE: Adverse event; AIMS: Aortic Irbesartan Marfan Study; ANCOVA: Analysis of covariance; AR: Adverse reaction; ARB: Angiotensin receptor blocker (also known as angiotensin II receptor antagonist); AT1: Angiotensin II receptor type 1; AT2: Angiotensin II receptor type 2; BMS: Bristol-Myers Squibb; BSA: Body surface area; CT: Computed tomography; CTA: Clinical trial authorisation; CTEU: Clinical Trials and Evaluation Unit; DMC: Data Monitoring Committee; DSMC: Data and Safety Monitoring Committee; ECG: Electrocardiography; eCRF: Electronic case record form; EDTA: Ethylenediaminetetraacetic acid; EudraCT: European Union Drug Regulating Authorities Clinical Trials; IMP: Investigational medicinal product; IVRS: Interactive voice recognition system; MFS: Marfan syndrome; MHRA: Medicines and Healthcare Products Regulatory Agency; MMP: Matrix metalloproteinase; MRC: Medical Research Council; MRI: Magnetic resonance imaging; NHS: National Health Service; NIH: National Institute of Health; NIHR: National Institute for Health Research; NRES: National Research Ethics Service; OD: Once daily; RCT: Randomized clinical trial; REC: Research Ethics Committee; SAE: Serious adverse event; SAR: Serious adverse reaction; SDV: Source data verification; SmPC: Summary of product characteristics; SUSAR: Suspected unexpected serious adverse reaction; TGF-β: Transforming growth factor beta; TEE: Transesophageal echocardiography; TSC: Trial Steering Committee; TTE: Transthoracic echocardiogram; UAR: Unexpected adverse reaction.

## Competing interests

The authors declare that they have no competing interests

## Authors’ contributions

MM (Chief Investigator) developed the study, participated in the design and co-ordination, and helped to draft the manuscript; grant holder (British Heart Foundation). AC developed the study, participated in the design and co-ordination, and helped to draft the manuscript; grant holder (British Heart Foundation). MF developed the study, participated in the design and co-ordination, and helped to draft the manuscript; grant holder (British Heart Foundation). XYJ developed the study, participated in the design and coordination, leads the echo core laboratory, and helped to draft the manuscript; grant holder (British Heart Foundation). WN participated in the design of the pharmacogenetics sub-study and helped to draft the manuscript. GE participated in the design of the study and helped to draft the manuscript. DG participated in the design of the TGF-β sub-study and sample core laboratory. OV assisted in the design of the study and sample core laboratory. WB participated in the statistical design and critical review of the manuscript. CF participated in the design and co-ordination of the study and helped to draft the manuscript. All authors read and approved the final manuscript.

## Supplementary Material

Additional file 1 Appendices. Click here for file
