# Supplementary material for: A prospective, randomized, placebo-controlled, double-blind, multicenter study of the effects of irbesartan on aortic dilatation in Marfan syndrome (AIMS trial): study protocol
Source: Trials. 2013 Dec 1;14:408. doi: 10.1186/1745-6215-14-408 (PMC4220813; doi:10.1186/1745-6215-14-408)
Supplement: Additional file 1 — Appendices. [file 1745-6215-14-408-S1.doc]

**Appendices**

**Echocardiography Protocols**

**1. General consideration of echocardiographic assessment:**

(1). Patients will be seen in centres that have routine access to latest echo scanning system (generally within 5 years) for recording trans-thoracic echo with standard M-mode, two dimensional and Doppler echocardiograms.

(2) Echo measurements will be taken by trans-thoracic echocardiograms performed at baseline (before the treatment) and annually for three to five years in order to assess the change of aortic dilatation. The echo study will be performed by experienced echo-cardiographer, according to a standardised protocol published by American Echo Society (ASE 2003). Each image section should include at least five cardiac beats for patients in sinus rhythm and seven to ten cardiac beats for non-sinus rhythm.

(3). An echo training session will be provided to participating staff from each centre on the standardised protocol prior to commencing the trial. This will facilitate echo image acquisition, protocols compliance, and off-line image analysis being consistently integrated for assuring a high quality data set.

The echo images are recorded in digital format and copied in DICOM Format to Magnetic Optical Disc or DVD for image transfer to the Echo Core Lab for a centralised reading at the John Radcliffe Hospital in Oxford. At the Echo Core Lab, a single experienced investigator (Dr XY Jin, who is blinded to Irbesartan prescription) will analyse all the echocardiograms according to a standardised protocol (see below) to reduce inter-observer variability and eliminate any bias of the treatment. According to American Echo Society guideline of Echo Core Lab for Clinical Trial (ASE 2009), a standardised management protocols is applied. In order to assess the reproducibility of echo image acquisition and image analysis, 5 percent of echo scans (in selected centre where it is practical ) and echo image analysis at the Core Lab will be randomly selected for repeating echo scan in one week and repeating image analysis in 3 months after initial scan or reading, respectively. The echo core lab will provide feedback to individual clinical centre regarding the image quality and protocols compliance.

**2. Key Echo data required for the study:**

The primary Echo endpoints of the AIMS trial are based on the absolute changes in aortic root diameter during the study course. Aortic root diameter will be measured at the annulus, in the sinuses of Valsalva at the tip of the open cusps at ninety degrees to the direction of flow, the sinotubular junction, ascending aorta, aortic arch and descending aorta. The most important aortic diameter is at the sinus of Valsalva.

**Secondary endpoints**

- Change in z score per year, where the z score is calculated on aortic sinus diameter and indexed to body surface area (BSA).
- Left ventricular function determined by end diastolic volumes and ejection fraction.
- Left ventricular muscle mass measurements by ASE formula.
- Heart valvular function.
- Aortic distensibility at sinus and ascending aorta.
- Aortic root geometry index
- ECG rhythm, LV voltage, QRS duration, QT interval and ST-T quantification.

**3. Echo image section & recording sequence: Minimal 5 cardiac beats at each image section**

(1). Parasternal Long axis View:

- This is the most important image section for assessing aortic root diameters. The maximal diameter of aortic root and ascending aorta must be imaged and recorded by interrogating the transducer.

(Zoomed image on aortic root) – for measuring LVOT, aortic annulus, Sinuses of Valsalva, Sino-tubular junction (figure 1).

- M mode through aortic sinus (zoomed image), then through sinus-AV / LA

-M-mode of LV at the tip of MV leaflet for measuring LV EDD, ESD, PW and IVS thickness. Again, this is one of the most important image section for determining LV function and LV mass

- CFM of MV and AV to detect any MR and AR

(2). Modified Parasternal Long Axis View: (more superior / lateral view, showing more of ascending aorta - optimise depth / gain / focus) - 2D measurements Sinotubular junction & proximal & distal ascending aorta. Follow this view up to see fullest extent of aorta.

M-Mode of ascending aortic diameter for determining distensibility along with blood pressure.

(3). Parastenal Short Axis View:

Sweep through from AV to apex & side to side to show TV / PV

- Concentrate on MV; CFM of AV, PV and TV

(4). Apical Four Chamber View: For LV EDV, ESV, LVEF

- 2D imaging showing LV, LA, RV, RA, MV & TV

- CFM of MV, TV

- PW Doppler of MV, TV

- CW Doppler of MR, TR

-PW tissue Doppler to measure longitudinal motion of MV annulus, Septum & TV annulus

(5). Apical Two Chamber View: - 2D LV / LA / MV / length of descending thoracic aorta

- 2b Measurement - descending thoracic aorta

(6). Apical Five Chamber View: for aortic valve PW and CW Doppler and CFM

- PW Doppler of LVOT

- CW Trans-aortic velocity +/ AR

- CFM of LVOT & AV for AR

(7). Supra Sternal View: - imaging of arch

- measurement of apex of arch

- CFM and PW Doppler of descending aorta

(8). Transverse Abdominal Scan: – 2D transverse cut through IVC / Aorta

(9). Longitudinal Abdominal Scan: 2D length of upper abdominal aorta

- Measurement of diameter of abdominal aorta at level of SMA

**4. Illustrations of Key Echo Measurements**

(1). Aortic Root Diameters (figure 1) measurement is based on Inner Edge to Inner Edge technique (the American Echo Society method) from 2D Echo image at end diastole (the onset of ECG q wave) and early-systole (when aortic cusp is fully opened).

Aortic root geometry will be determined by the diameter ratio of aortic sinus to annulus, and to sino-tubular junction; ascending aortic diameter to annulus, and sino-tubular junction diameter, respectively, at end diastole and mid-systole.

Aortic root distensibility: determined by systolic changes in M-mode aortic diameter at sinus and ascending aorta, along with systemic pulse pressure

(3). Regurgitation of aortic valve

Severity: semi-quantitative assessment (none/trivial (0), mild (+1), moderate (+1), or severe(+3), using the ratio of AR jet width to LVOT diameter at <0.10, 0.10-0.24, 0.25-0.40, >0.40.

(4). LV chamber dimensions, LVMI and LVEF

Assessing patient’s left ventricular function, i.e. chamber sizes, septal thickness, wall thickness, will use 2D/M-mode measurements. Thus LVMI and systolic function can be determined.

The following variables will be obtained by 2-D guided M-mode and measured according to the criteria of the American Society of Echocardiography.

- Interventricular septal thickness at end diastole in mm
- Left ventricular internal diameter at end diastole in mm
- Left ventricular posterior wall thickness at end diastole in mm
- Left ventricular internal diameter at end systole in mm
- Interventricular septal thickness at end systole in mm
- Left ventricular posterior wall thickness at end systole in mm
- Left atrial dimension at systole in cm

(5). M-mode Recordings: M-mode recordings of the left ventricle, left atrium, and aortic root should be obtained from the parasternal window using 2-D guided beam alignment. Structures should be measured according to the methods recommended by the American Society of Echocardiography. If M-mode recordings are technically unsatisfactory, measurements can be made from 2-D images.

(6). Left ventricular ejection fraction: Using 2-D imaging, LVEF will be assessed by quantitative methods. The Modified Simpson’s rule (Method of Discs) is the recommended quantitative method. However, the Quinones method may also be used if endocardial definition is technically difficult.

**5. Echo Quality Assurance Procedures**

(1). Study Centres

All protocol-required echocardiograms should be performed at the study centre only. Patients must be able to return to the study centre for protocol required follow up of echocardiograms.

Each centre will have a designated echo-cardiographer responsible for the conduct of echocardiography, review of image quality and copying the whole study in digital format (DICOM), who will also serve as a contact point for communications with echo core lab (The echo-cardiologist at study centre may serve in this role if they personally perform the exams). Centres should limit the number of echo-cardiographers (no more than two) performing the protocol-required exams but should have enough capacity to cover periods of absence at their institution.

(2). Echo Protocol compliance

Centres are expected to follow the echo protocol in the standardised manner as described above to facilitate review by the Echo Core Lab.

Standardised echo tracking form will be used to eliminate variation in the basic clinical and administrative information required for the echo core lab.

(3). Echo Core Lab

The development of echocardiographic protocols and the running of Core Echo Lab are led by the lead Echo-Cardiologist of the AIMS trial (Dr XY Jin). The roles of the core lab echo-cardiologist are as follow:

- Provide direction to the development of methods for data acquisition, measurement, and interpretation.
- Review the study echo image quality, analyse echo images submitted from each centre, comment on each centre’s compliance to echo protocols.
- Work with the study centres to resolve any data quality issues or discrepancies that are identified through the reviews.
- Provide the echo data analysis and quality assurance.

**Echocardiographic Derivation formulas**

1. BSA and Z score calculations:

Body surface area (BSA) is calculated using the Dubois and Dubois formula: BSA (m^2^) = 0.007184 x H^0.725 x W^0.425 with H height in cm, W weight in kg.

Predicted root sinus diameter is calculated using Cornell data-based formulae.

Age up to 19: 10.2 + 9.8*BSA, SD = 1.8 mm.

Age 20 to 40: 9.7 + 11.2*BSA, SD = 2.4 mm.

Z-score is (measured root diameter - predicted root sinus diameter) / SD

2. Mean Pressure Gradient (Δ PMean Ao) Across the Aortic Valve in mmHg

Δ PMean Ao = MGV2 - MGV1

Where: MGV2 is the mean pressure gradient* across the aortic valve in mmHg, and MGV1 is the mean pressure gradient* from the left ventricular outflow tract in mmHg.

*Measured with the ultrasound system analysis software

3. Peak Pressure Gradient (Δ PPeak Ao) Across the Aortic Valve in mmHg

Δ PPeak Ao = 4 x (V22 - V12)

Where:V2 is the peak velocity across the aortic valve in m/sec*, and

V1 is the peak velocity from the left ventricular outflow tract in m/sec*

*Velocities measured in cm/sec will be converted to m/sec

4. Cross-Sectional Area (CSA) of the Left Ventricular Outflow Tract in cm2

CSA = (LVOTdiam)2 x 0.785

Where: LVOTdiam is the left ventricular outflow tract diameter in cm

6. Stroke Volume (SV) in ml/beat

SV = CSA x TVILVOT

Where: CSA is the cross-sectional area of the left ventricular outflow tract in cm2, and

VTILVOT is the velocity time integral from the left ventricular outflow tract in cm

7. Cardiac Output (CO) in l/min

CO = (SV x HR)/1000

Where: SV is the stroke volume in ml/beat, and

HR is the heart rate in beats per minute

8. Cardiac Index (CI) in l/min/m2

CI = CO/BSA

Where: CO is the cardiac output in l/minute, and

BSA is the body surface area in m2

9. Aortic Effective Orifice Area (EOAA) in cm2

EOAA = CSA x (VTILVOT/VTIAPros)

Where: CSA is the cross-sectional area of the left ventricular outflow tract in cm2, VTILVOT is the velocity time integral of the left ventricular outflow tract in cm, and VTIAv is the velocity time integral of the aortic valve in cm.

10. Indexed Effective Orifice Area (Ind EOAA) for Aortic valve in cm2/m2

Ind EOAA = EOAA/BSA

Where: EOAA is the effective orifice area for the aortic valve in cm2, and

BSA is the body surface area in m2

11. Aortic Regurgitant Jet Area/Left Ventricular Outflow Tract Area (RJAAo/ALVOT) ratio in%

RJAAo/ALVOT Ratio = RJAAo/ALVOT x 100

Where: RJAAo is the area in cm2 of the aortic regurgitant jet at its origin in the short axis view, and ALVOT is the area in cm2 of the left ventricular outflow tract in the short axis view.

12. Left Ventricular Mass (LVM) in grams.

LVM = 0.83 x [(LVIDD + LVPW + IVS)3 - (LVIDD)3] + 0.6

Where: LVIDD is the left ventricular internal dimension at end diastole in cm measured by mode, LVPW is the left ventricular posterior wall thickness at end diastole in cm measured by M-mode, and IVS is the inter-ventricular wall thickness at end diastole in cm measured by M-mode

13. Left Ventricular Mass Index (LVMI) in g/m2 body surface area

LVMI = LVM/BSA

Where: LVM is left ventricular mass in g, and

BSA is body surface area in m2

**Appendix 2.0**

**Expected drug related adverse reactions**

Please refer to the Summary of Product Characteristics as provided.

Side effects of Irbesartan from the hypertension clinical trials are listed below. The overall incidence of adverse reactions did not differ between the Irbesartan and placebo groups, and was not related to dose, gender, age, race or duration of treatment.

Very common: Hyperkalaemia (a higher rate in diabetic patients treated with Irbesartan compared with placebo).

Common: [1] Significant increases in plasma creatine kinase were observed in patients treated with Irbesartan although these were not associated with identifiable clinical musculoskeletal events. [2] Dizziness, orthostatic dizziness, [3] nausea/vomiting, [4] musculoskeletal pain.

Uncommon: [1] tachycardia, [2] cough, [3] diarrhoea, [4] dyspepsia/heartburn, [5] flushing, [5] chest pain, [6] sexual dysfunction

Other post marketing adverse reactions include; [1] headache, [2] tinnitus, [3] dysgeusia, [4] impaired renal function, [5] leukocytoclastic vasculitis, [6] athralgia/myalgia/muscle cramps, [7] angioedema/rash/uticaria, [8] hepatitis/abnormal liver function.

Side effects in 318 hypertensive paediatric patients between 6 and 16 years treated over 3 weeks included headache (7.9%), hypotension (2.2%), dizziness (1.9%), cough (0.9%). Over a 26 week open label treatment period, adverse reactions included creatinine increases (6.5%) and elevated CK values (2%).
